# Online Focus Group Discussion is a Valid and Feasible Mode When Investigating Sensitive Topics Among Young Persons With a Cancer Experience

**DOI:** 10.2196/resprot.5616

**Published:** 2016-05-09

**Authors:** Lena Wettergren, Lars E Eriksson, Jenny Nilsson, Anna Jervaeus, Claudia Lampic

**Affiliations:** ^1^ Division of Nursing Department of Neurobiology, Care Sciences and Society Karolinska Institutet Huddinge Sweden; ^2^ Medical Management Center (MMC) Department of Learning, Informatics, Management and Ethics (LIME) Karolinska Institutet Stockholm Sweden; ^3^ Department of Infectious Diseases Karolinska University Hospital Stockholm Sweden; ^4^ School of Health Sciences City University London London United Kingdom

**Keywords:** adolescent, data collection, focus groups, Internet, neoplasms, young adult

## Abstract

**Background:**

Clinical research often lacks participants of young age. Adding to the small amount of scientific studies that focus on the population entering adulthood, there are also difficulties to recruit them. To overcome this, there is a need to develop and scientifically evaluate modes for data collection that are suitable for adolescents and young adults. With this in mind we performed 39 online focus group discussions among young survivors of childhood cancer to explore thoughts and experiences around dating, being intimate with someone, and having children.

**Objective:**

The aim of the study was to evaluate online focus group discussions as a mode for data collection on sensitive issues among young persons with a cancer experience.

**Methods:**

One hundred thirty-three young persons (16-25 years) previously diagnosed with cancer, participated in 39 synchronous online focus group discussions (response rate 134/369, 36%). The mode of administration was evaluated by analyzing participant characteristics and interactions during discussions, as well as group members’ evaluations of the discussions.

**Results:**

Persons diagnosed with central nervous tumors (n=30, 27%) participated to a lower extent than those with other cancer types (n=103, 39%; χ 2= 4.89, *P*=.03). The participants described various health impairments that correspond to what would be expected among cancer survivors including neuropsychiatric conditions and writing disabilities. Even though participants were interested in others’ experiences, sexual issues needed more probing by the moderators than did fertility-related issues. Group evaluations revealed that participants appreciated communicating on the suggested topics and thought that it was easier to discuss sex when it was possible to be anonymous toward other group members.

**Conclusions:**

Online focus group discussions, with anonymous participation, are suggested to be a feasible and valid mode for collecting sensitive data among young persons with a cancer experience.

## Introduction

Focus group discussions is an established mode for collecting data that have the possibility to, in some ways, move beyond individual interviews by simultaneously taking different perspectives and opinions into account when letting participants interact during a moderated discussion [1,2]. Such discussions may also be performed online [3], which can increase response rates in groups comfortable using computers (eg, young populations) [4].

Based on the abovementioned, we performed online focus group discussions with young childhood cancer survivors to explore their thoughts about fertility and sexuality. The aims were to investigate what adolescent and young adult survivors of childhood cancer think about the risk of being infertile and how they reason about having biological children [5]. Additionally, we aimed to explore this group’s views about sex and sexual experiences and their possible needs for care and support from health care professionals regarding sexual life [6]. The transcripts from the group discussions were analysed inductively with content analysis [7]. The risk of infertility was viewed to negatively impact on well-being and intimate relationships [5]. The findings regarding sexuality showed that many participants had not reflected over the possibility that their cancer experience could impact on sexual life [6]. Still, thoughts and worries were expressed, such as feeling insecure and not keeping up with your peers. Physical complaints included vaginal dryness, difficulties getting and keeping erections, and reaching orgasm.

While online focus group discussions may facilitate discussion of sensitive issues [4], advantages and disadvantages of this mode of data collection in vulnerable populations (eg, patients) are largely unknown [8]. The aim of the present study was therefore to evaluate online focus group discussions as a mode for data collection on sensitive issues among young persons with a cancer experience.

## Methods

The main study’s procedure and aims have briefly been presented in the introduction. This paper will evaluate the mode of administration (ie, online focus group discussions).

### Participants

Four hundred young persons, 16-24 years old, and 5 years or more beyond a childhood cancer diagnosis, were identified through the Swedish Childhood Cancer Registry. Diagnoses were selected based on their potential negative impact on fertility: Hodgkin´s lymphoma, Ewing/Ewing-like sarcoma, osteosarcoma, rhabdomyosarcoma, neuroblastoma, and tumors of the central nervous system (CNS). The register’s total population of persons with solid tumors in the age range of focus, except tumors of the CNS, was approached (N=280). As the number of persons treated for tumors of the CNS was large, a random sample was selected (n=120 from the total sample). Thirty-one persons were excluded due to self or parent-reported cognitive disabilities (n=7), other disabilities (n=1), not being possible to reach at a Swedish address (n=19), deceased (n=1), or other reasons, such as undergoing cancer treatment (n=3). Among the remaining 369 eligible participants, 36% (134/369) accepted participation. One discussion included only one participant and was not included in the analysis why the results are based on 133 participants.

### Procedure

Ethical approval was obtained from the Regional Ethical Review Board in Stockholm. Potential participants received a letter with information about the study; voluntariness and confidentiality were stressed. Written informed consent was obtained from all participants.

### Data Collection

Focus group discussions were performed through an existing chat platform developed together with an Internet consultancy company [9]. Thirty-nine discussions were conducted with two to five participants in each group. Group discussions were performed synchronously and lasted for approximately 90 minutes (range, 65-130). Each group was typically led by two moderators with backgrounds in cancer care, pediatric care, midwifery, and/or psychology. Those who had signed up for a focus group discussion received login details by text message or phone before start of the discussion. The platform allowed the informants access from a computer at any location, using an alias. In this way, participants could be anonymous toward each other while not in relation to the moderators. It was, however, not uncommon that participants chose her/his real name as alias. An effort was made to mix sexes and to have similar ages in the groups. Directly after participation, each participant was invited to anonymously report their experiences from participating in the study in a separate chat forum by answering five items with fixed-response alternatives and four questions with an open response format.

### Analysis

The advantages and disadvantages with the mode of data collection was studied in three ways. We analyzed characteristics of those who participated, interactions during discussions, and the participants’ evaluation of the focus group discussions.

## Results

### Who Participated?

The median age of participants was 21 ranging from 16 to 25 (interquartile range 4); self-reported relationship status and sexual experience as disclosed during group discussions are presented in Table 1. All but 4 of 39 conducted groups had mixed sexes. The response rate was higher among those diagnosed with solid tumors than among those diagnosed with CNS-tumors (n=103, 39% vs. n=30, 27%; χ^2^= 4.89, *P*=.03). Apart from sexual problems and fertility-related concerns, participants mentioned various health impairments such as being amputated, fatigued, depressed, and having cognitive difficulties. However, as health was not the focus of this study, we do not know if the mentioned health problems were related to sexuality or fertility.

**Table 1:** Characteristics of participants.

Self-reported situation		Total	Females	Males
		n=133 (%)	n=67 (%)	n=66 (%)
**Relationship status**				
	Partner relationship	48 (36)	28 (42)	20 (30)
	Dating/flirting	5 (5)	3 (5)	3 (5)
	Single	62 (47)	28 (42)	34 (52)
	Not reported	17 (13)	8 (12)	9 (14)
**Sexual experience**				
	Have sexual experience	103 (77)	58 (87)	45 (68)
	No sexual experience	16 (12)	3 (5)	13 (20)
	Not reported	14 (11)	6 (9)	8 (12)

All of those who signed up to participate in a group also showed up and almost all of them who started in a group discussion stayed through the whole discussion. Some participants spontaneously declared that they had writing disabilities, which also was obvious in their spelling and grammar. A few, on their own initiative, disclosed that they had a neuropsychiatric disorder such as Asperger’s and still, they reported the chat format as feasible. Participants who used an alias, possible to identify as a gendered name, never explicitly expressed having a relationship with someone of the same sex but the opposite was common (ie, heterosexual relationships). Moderators used gender-neutral expressions (eg, partner) when discussing partner relationships.

### Were Sensitive Issues Discussed and How Did Participants Interact With Each Other?

Sexual issues needed more probing by the moderators than did fertility-related issues. However, when sex was brought up on the agenda, the issue was discussed. Communication between participants in the group discussions was overall respectful and supportive. Participants encouraged each other to take steps in their lives if they considered something problematic (eg, to meet someone or try a different approach). Different views were often expressed but there were seldom clear disagreements [6]. Participants were curious and asked each other about age, diagnosis, and sometimes where in the country they had received their treatment. Some of them identified themselves and agreed to continue chatting afterward on Facebook.

### Participants’ Evaluation of the Online Discussions

Directly after participation, group members were invited to anonymously report their experiences in a Web-based survey which 50% (67/134) chose to do. Almost all participants who answered the evaluation experienced their participation as overall positive, and a majority reported that it was easier to discuss when you were anonymous, and that the moderators stimulated the chat (Table 2). Participants’ responses to the open questions revealed positive experiences of chatting with others with similar experiences and expressed that the online format made it possible to be anonymous which facilitated sharing of sensitive information. Suggestions for improvement included more developed discussion topics, a higher speed in the discussions, not having discussions with too few participants (ie, 2), and having longer or repeated discussions (Textbox 1).

**Table 2:** Participants’ evaluation of the chat discussions (n=67).

	Highly agree	Somewhat agree	Do not agree
	n (%)	n (%)	n (%)
Overall positive experience	60 (91)	6 (9)	0
Possibility to express yourself^a^	58 (87)	7 (10)	0
The web hindered the discussion^a^	5 (8)	7 (10)	54 (81)
Anonymity made it easy to discuss^a^	38 (57)	10 (15)	17 (26)
Did moderators stimulate the chat?^a^	48 (72)	16 (24)	1 (<1)

^a^Due to missing answers percentages do not reach 100% for all questions

**Examples of participants’ answers on the free text items in the evaluation form.**

What information would have been difficult to communicate face-to-face?

- *Issues around sex and things like that maybe*
- *Maybe you do not dare to say what you want in a group if you aren’t anonymous*
- *To talk about sex and relations is easier behind a screen*

What was good?

- *To hear what others feel and think. The anonymity made it possible to be honest and you could be at home without spending too much time*
- *The anonymity and the internet chat idea was very good, it made it possible to write things you wouldn’t dare to share otherwise*

What was bad?

- *I thought the discussion leaders were a little unclear with some questions. I also thought the issues were a bit ’fluffy’ and that only 2 persons really discussed*
- *A little slow at times*
- *The time was a bit short*

Do you have any suggestions for improvement?

- *A little more tempo*
- *Have longer time or repeated chat forums*
- *More participants in the chat*

## Discussion

### Advantages with the Mode

Online focus group discussions, performed with the possibility for participants to be anonymous toward each other, was shown to be a feasible mode for collecting sensitive data among young persons treated for cancer during childhood. Both persons with and without health problems participated in the group discussions. The lower response rate seen for participants diagnosed with CNS tumors may indicate that this mode of data collection is less suitable for certain groups. Still, persons with self-reported cognitive impairments signed up and participated in group discussions and this did not generate problems.

The study partly used a random sampling procedure not typical for these kinds of studies. Without a purposeful sampling technique you risk including persons that may have difficulties to communicate that can result in a less interactive dialogue. However, we did not experience this, which may reflect the fact that we had experienced moderators, preferably two per group, who carefully followed all group members through every discussion. Nevertheless, we recommend the number of participants in online focus group discussions, if conducted synchronously, to be at least three but not to exceed five.

### Relation to Previous Findings

The present study confirms previous findings showing that an online format meets the need of convenience commonly addressed by young cancer survivors [4] and may be advantageous for sensitive topics [10] in contexts with high access to computers and Internet [11]. Furthermore, in the present study, the possibility to use an alias to be able to be anonymous while chatting about sensitive issues was highlighted as positive by many participants.

Discussing sexual experiences in groups with mixed sexes was found to be feasible and appreciated in a Swedish context and, to our knowledge, not previously performed among cancer survivors. The approximate numbers of female and male participants who, during a group discussion, reported that they had sexual experiences with a partner are in line with figures for the general population of similar ages [12].

### Conclusion

Based on our findings, online focus group discussions are recommended for collecting data on sensitive topics among young people with various health deficiencies. This may be of great value when reaching out to populations who might be difficult to engage in face-to-face focus groups.

## Abbreviations

CNS: central nervous system
