# Metadata and Title Correction: Online Focus Group Discussion is a Valid and Feasible Mode When Investigating Sensitive Topics Among Young Persons With a Cancer Experience

**DOI:** 10.2196/resprot.5971

**Published:** 2016-05-12

**Authors:** Lena Wettergren, Lars E Eriksson, Jenny Nilsson, Anna Jervaeus, Claudia Lampic

**Affiliations:** ^1^ Division of Nursing Department of Neurobiology, Care Sciences and Society Karolinska Institutet Huddinge Sweden; ^2^ Medical Management Center (MMC) Department of Learning, Informatics, Management and Ethics (LIME) Karolinska Institutet Stockholm Sweden; ^3^ Department of Infectious Diseases Karolinska University Hospital Stockholm Sweden; ^4^ School of Health Sciences City University London London United Kingdom

The title of the paper originally entitled “Content Analysis of Online Focus Group Discussions is a Valid and Feasible Mode When Investigating Sensitive Topics Among Young Persons With a Cancer Experience” (JMIR ResProtoc 2016 May 9; 5(2):e86) was corrected to read “Online Focus Group Discussion is a Valid and Feasible Mode When Investigating Sensitive Topics Among Young Persons With a Cancer Experience”. The error was introduced by the editor in the final proofreading process to remove a grammatical issue, but the authors feel that “content analysis” in the title is incorrect because content analysis was not the method used for analyzing data in the present study. The advantages and disadvantages with the mode of data collection was judged by analyzing characteristics of those who participated, interactions during discussions and the participants’ evaluation of the focus group discussions. Secondly, there was also an incorrect affiliation footnote reading “null” associated with author Lena Wettergren, which has now been removed from the metadata. These errors have been corrected in the online version of the paper on the JMIR website on May 10 and May 12, 2016, respectively, together with publishing this correction notice.  There are no changes to the contents of the paper. A correction notice has been sent to PubMed. This was done before submission to Pubmed Central and other full-text repositories.

